# Facial expression at retrieval affects recognition of facial identity

**DOI:** 10.3389/fpsyg.2015.00780

**Published:** 2015-06-09

**Authors:** Wenfeng Chen, Chang Hong Liu, Huiyun Li, Ke Tong, Naixin Ren, Xiaolan Fu

**Affiliations:** ^1^State Key Laboratory of Brain and Cognitive Science, Institute of Psychology, Chinese Academy of SciencesBeijing, China; ^2^Department of Psychology, Bournemouth UniversityPoole, UK; ^3^University of Chinese Academy of SciencesBeijing, China

**Keywords:** facial expression, identity recognition, emotional cue, memory retrieval

## Abstract

It is well known that memory can be modulated by emotional stimuli at the time of encoding and consolidation. For example, happy faces create better identity recognition than faces with certain other expressions. However, the influence of facial expression at the time of retrieval remains unknown in the literature. To separate the potential influence of expression at retrieval from its effects at earlier stages, we had participants learn neutral faces but manipulated facial expression at the time of memory retrieval in a standard old/new recognition task. The results showed a clear effect of facial expression, where happy test faces were identified more successfully than angry test faces. This effect is unlikely due to greater image similarity between the neural training face and the happy test face, because image analysis showed that the happy test faces are in fact less similar to the neutral training faces relative to the angry test faces. In the second experiment, we investigated whether this emotional effect is affected by the expression at the time of learning. We employed angry or happy faces as learning stimuli, and angry, happy, and neutral faces as test stimuli. The results showed that the emotional effect at retrieval is robust across different encoding conditions with happy or angry expressions. These findings indicate that emotional expressions do not only affect the stages of encoding and consolidation, but also the retrieval process in identity recognition.

## Introduction

It is well known that memory can be modulated by emotion. Studies have often shown that certain emotional stimuli are more efficiently encoded, consolidated, and retrieved than neutral stimuli (Kensinger, 2004; LaBar and Cabeza, 2006; Righi et al., 2012). Memory for emotional stimuli, whether they are a list of words, scenes, or faces, is often more vivid and more accurate than for neutral stimuli (Hamann et al., 1999; Kensinger, 2004; Kensinger and Schacter, 2005; LaBar and Cabeza, 2006; Righi et al., 2012).

However, not all emotional stimuli are equally effective. Depending on research focus, different comparisons have been used to measure the effect of emotional stimuli. For instance, studies using non-face stimuli tend to compare effects of emotional with neutral stimuli without directly comparing effects of positive and negative stimuli (e.g., Mickley and Kensinger, 2008). While comparing effects of positive vs. negative expressions is common in face studies, it is often based on a single negative facial expression (e.g., anger, fear, or sad). Partly due to these drastically different choices of methods, the literature has often shown mixed results about the impact of emotional stimuli. Some studies reported better memory for faces with negative expressions (e.g., Sergerie et al., 2005, 2007; Righi et al., 2012), whereas others found superior memory for faces with happy expressions (e.g., Gallegos and Tranel, 2005; Shimamura et al., 2006; D’Argembeau and Van der Linden, 2007, 2011; Liu et al., 2014). This study is mainly concerned with the effect of facial expression on face memory.

In most of these studies, the participants were asked to recognize the identity of the learned faces in a test session where the test faces were often shown with a neutral expression. Based on their findings, most authors have suggested that processing of emotional expression plays a role at stages of encoding and consolidation (Phelps, 2004; Talmi, 2013). A modulation model has been proposed to explain the advantage of certain emotional stimuli (McGaugh, 2004; Schmidt and Saari, 2007), according to which emotional arousal during an encoding experience causes the activation of the amygdala to improve the consolidation of memory traces stored in hippocampus. This model provides a partial account for the effect of emotional memory. For example, Righi et al. (2012) found that happy and fearful faces elicited an enhanced Late Positive Potential (LPP) amplitude, which may reflect a privileged access to encoding processes and better memory consolidation. Likewise, a mediation theory (Talmi et al., 2007; Talmi, 2013) suggests that emotional stimuli recruit more attentional resources than neutral stimuli do during encoding. Research on the influence of face context seems to support this theory. For example, the emotional context surrounding a neutral face at encoding hampers recognition of isolated neutral faces (Van den Stock and de Gelder, 2012, 2014).

However, the effect of expression at retrieval is often neglected in these studies. It remains unknown whether emotional expression also has an influence on memory retrieval. Although some studies also used emotional expressions at test, they used identical emotional stimuli at learning and test. This makes it difficult to distinguish the effects of emotional content at the stages of retrieval and encoding. Can there be an effect of emotional retrieval cue when neutral faces are learned (i.e., when emotional effect at learning is absent)? This was one of the key research questions in the present study.

Given that memory is a function of the similarity between encoding and retrieval operations (Ritchey et al., 2013), the context at memory retrieval, as well as at initial encoding, is also important (Talmi, 2013). Memory retrieval involves the reactivation of neural states similar to those experienced during initial encoding, and varies as a function of encoding-retrieval match (Tulving and Thomson, 1973; Ritchey et al., 2013). There are at least two kinds of stimulus associations contributing to the reactivation based on encoding-retrieval correspondence: the perceptual and emotional associations (Danker and Anderson, 2010). Depending on research methods, the contributions from each are not always identifiable. Some studies employed the same face images as learning and test stimuli, and this makes it difficult to estimate the extent to which the retrieval performance depended on perceptual association (image similarity) or emotional association (emotional similarity). Some studies used emotional faces in a learning session but neutral faces in the test session. These studies suggest that face recognition is modulated by emotional information at encoding (McGaugh, 2004; Schmidt and Saari, 2007; Righi et al., 2012). However, physical similarity between emotional expression and neutral expression may also account for the advantage of an emotional expression on identity recognition. For example, relative to happy expression, negative expressions may be more similar to neutral expression (Chen et al., 2011), leading to a stronger perceptual association. Furthermore, given that reactivation of the regions involved in processing the stimulus at encoding is difficult to be distinguished from activation of such regions caused by attentional or perceptual or emotional processing of the same stimulus at retrieval (Danker and Anderson, 2010), it is difficult to separate the independent emotion effect at retrieval from that at encoding and consolidation.

To tackle these issues, the present study investigated the effect of facial expression at the stage of retrieval and whether any effect of this has to rely on emotional association between the learning and test face stimuli. Apart from the baseline conditions, where the face stimuli at learning and test were identical, the facial expression used at learning formed no emotional association with the face stimuli at test in our experiments. We investigated whether seeing an emotional face at the test session affects retrieval of the faces studied with a neutral expression (Experiment 1) or emotional expression (Experiment 2). We studied how positive and negative facial expressions in the test stimuli impact on memory retrieval differently. Each learned face was tested with one of three expressions: happiness, anger, or neutral.

We predicted a happy-expression advantage because the happy expression has been shown to produce better identity recognition memory than some other expressions (see Liu et al., 2014, for a review). Although previous results of the happy-expression advantage are rather mixed, it should be noted that they are based on the information before retrieval, where mechanisms for a happy expression advantage may be quite different. In the critical condition of the present study, we used a happy face as a retrieval cue to access and match the stored face identity representation. We were interested whether this could facilitate the retrieval process. The prediction of happy-expression advantage is consistent with the observation that positive emotions facilitate whereas negative emotions impair holistic face processing (Curby et al., 2012). Given that holistic processing is essential for superior face recognition, a happy face as a retrieval cue should be more effective for performing a search of the stored target face identity.

However, since the similarity between face images used for encoding and retrieval is also an important predictor of performance, we assessed how the physical similarity contributes to the effect of emotional cues at retrieval. That is, we tested the extent to which image similarity affects identity recognition. We expected best performance for the baseline condition, where face images at the learning and test sessions were identical. The key research question for us, however, was whether performance for different learning and test images also depends on image similarity. To this aim, we measured physical similarity between face images with neutral and happy (or angry) expressions. Better performance can be expected if there is a greater image similarity between neutral and angry expression than between neutral and happy expression. We tested the possibility that face recognition may be more influenced by processing of emotional information than by physical image similarity. For example, it is possible that although there is a greater similarity between images of neutral and angry expression than between neutral and happy expressions, test faces with a happy expression would still produce a better performance than those with an angry expression.

There is evidence that processing of emotional information at the stages of encoding and consolidation can influence the different aspects of memory. Mickley and Kensinger (2008) speculated from the neural data that memories for negative items are recollected more vividly, whereas positive stimuli tend to create feelings of familiarity. However, their behavioral data did not show a significant difference between the effects of positive and negative pictures on familiarity and recollection. Others have reported that face memory is more often correctly associated with “remember” responses when a previously studied happy face (rather than an angry expression) is shown with a neutral expression in the test session (D’Argembeau et al., 2003). In addition, there is evidence that emotional information at encoding and consolidation can modulate the response bias. For example, negative stimuli are more likely to be classified as old (i.e., a liberal bias), whereas positive stimuli are prone to be classified as new (i.e., a conservative bias; Maratos et al., 2001; Windmann and Kutas, 2001; Sergerie et al., 2007). Contradictory finding has also been reported. For example, Phaf and Rotteveel (2005) found that positive stimuli created a more liberal recognition bias, whereas negative stimuli a more cautious bias (i.e., the tendency to classify negative stimuli as new). Hills et al. (2011) suggest that negative emotion (e.g., sadness) may encourage more elaborate processing, which leads to increased arousal and tendency to respond with an old response. However, it remains unclear which dimensions of emotion lead to these different response biases.

Because positive and negative emotions may have different effects on recollection and familiarity judgment (e.g., D’Argembeau et al., 2003), we examined the effect of facial expression on both these aspects of memory. Recollection is a retrieval of details associated with previously experienced stimuli, and familiarity is the feeling that the stimulus was previously seen but lacking details of recollection. A common way to distinguish these cognitive processes is to ask participants whether they are able to vividly “remember” the item or simply “know” that it was presented because it looks familiar. To investigate whether emotional expression has a similar pattern of effect for memory retrieval and to identify the source of positive-negative difference, we also applied the remember/know task in the present study. Following prior research we also assessed the effect of expression on response bias.

## Experiment 1

The main purpose of this experiment was to assess the effect of facial expression at the stage of retrieval. Participants were asked to learn faces with a neutral expression. At the test session, two thirds of the learned faces were shown with an emotional expression (happy or angry), whereas the remains were shown in the same neutral expression. We assessed whether a test face with the happy expression is more effective for memory retrieval than that with the angry expression. Since the faces were learned with a neutral expression, the manipulation ruled out the possibility that any effect of emotional expression was created at the stages of encoding and consolidation.

### Method

#### Participants

Fourteen undergraduate students from Chinese Agricultural University (mean age 23.7 years, SD = 1.7, six females) participated in this experiment for a small payment. All had normal or correct-to-normal vision. The study was approved by the Institutional Ethics Review Board of the Institute of Psychology, Chinese Academy of Sciences. All participants were treated in accordance with the APA’s guidelines. Informed consent was obtained from each participant prior to the experiment.

#### Materials

The face stimuli were taken from the face pool developed by the authors and the CAS-PEAL Large-Scale Chinese Face Database (Gao et al., 2008). We used 216 Chinese models in six experimental blocks, in additional to 36 models in a practice block. Each face model was shown against a gray background with three expressions: happy, angry, and neutral. The faces were converted to gray-level images. To minimize the low-level image cues for the task, the luminance and root-mean-square contrast of the images were scaled to the grand means.

Following Adolphs et al. (2008), and Chen et al. (2011), we computed the physical similarity between images at learning and test for each facial expression using the Structural SIMilarity index (SSIM; https://ece.uwaterloo.ca/~z70wang/research/ssim/). The SSIM score is a quantitative estimate of the similarity between two images that corresponds closely to similarity judgments by human observers (Adolphs et al., 2008). Its scores range from –1 (entirely different) to 1 (identical). The SSIM score for any pair of identical images is always 1. Because the paired images in the neutral condition were identical, we only calculated the image similarity for conditions that involved different images. Results showed the mean similarity scores for happy–neutral pairs and angry–neutral pairs were 0.62 (SD = 0.06) and 0.64 (SD = 0.07), respectively. Both scores were significantly different from 1 (identical neutral pairs), *t’*s(215) > 136.54, *p’*s < 0.001. Happy–neutral pairs were less similar than angry–neutral pairs, *t*(215) = 5.77, *p* < 0.001.

#### Design

We employed a within-participant design. The independent variable was test expression (happy, angry, and neutral).

#### Procedure

The experiment was run in six experimental blocks following a practice block. Each block consisted of 36 models with half as target stimuli and half as lure stimuli at test. Each block consisted of three sequential stages: learning, filler, and test.

The learning stage consisted of 18 trials. Each trial began with a 500 ms central fixation cross, followed by a learning face presented for 5 s in the center of the screen. Participants were instructed to remember these faces.

The filler stage consisted of a 5-min arithmetic task where participants performed simple two-digit addition and subtraction computations. Following this, they completed a memory retrieval test in which they viewed 36 faces (18 targets and 18 lures) sequentially presented on the screen. The test face appeared for 3 s after a 500 ms fixation cross. Participants were instructed to judge whether each face identity had been previously presented (old/new judgment). They were told to give their answer as quickly and accurately as possible by pressing one of the two keys labeled ‘Yes’ or ‘No.’ One-third of the old faces in this test stage were shown with a happy expression, one-third with an angry expression, and the remains with a neutral expression. The same number of each expression was applied to the new faces. When participants recognized a face as an old one, they were asked to further indicate whether they were able to vividly “Remember” the face, or simply “Know” the face because it looked familiar, or whether they were simply making a “Guess.” The three options were referred to as a RKG judgment.

### Results and Discussion

We calculated *d′* scores for each participant based on the hit (H) and false alarm (FA) rates, where *d′* = z(H) – z(FA) (Stanislaw and Todorov, 1999). *d′* is a parametric measure of sensitivity that indicates how well a participant discriminates targets from lures.

**FIGURE 1:**
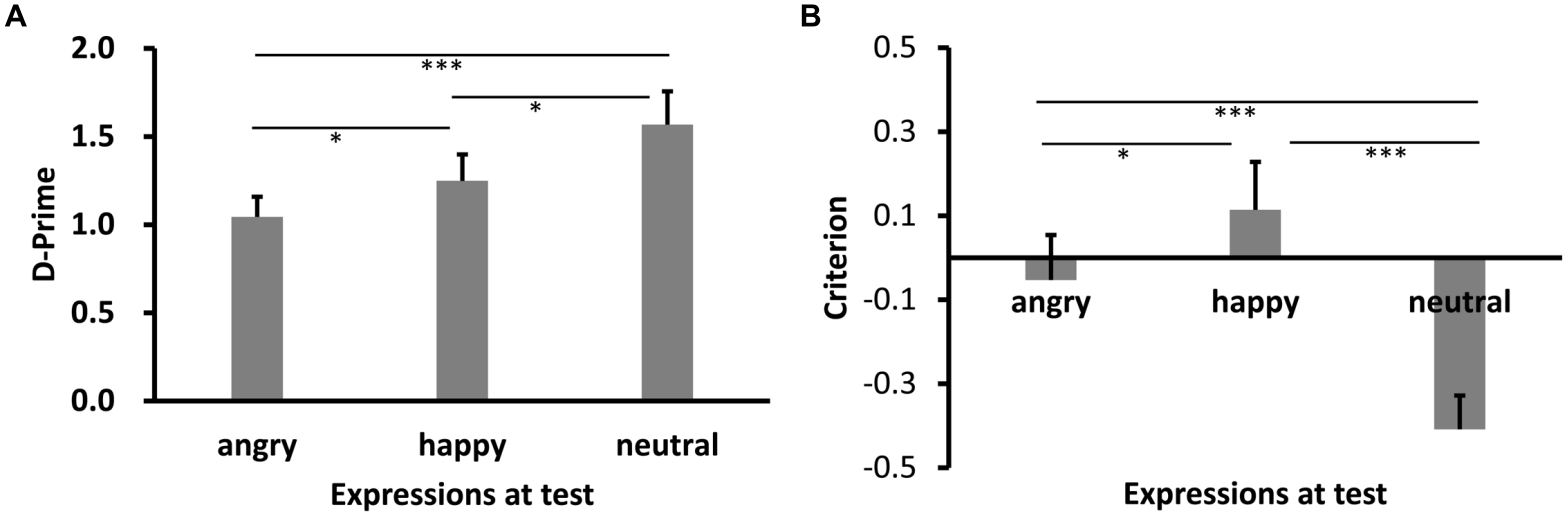
**Sensitivity *d*′ (A) and Criterion c (B) results as a function of test expression (Experiment 1)**. Error bars represent 1 SEM. ^∗^*p* < 0.05;, ^∗∗∗^*p* < 0.001.

The mean *d′* results across participants are shown in **Figure 1A**. Shapiro–Wilk tests showed that the data in all conditions were normally distributed (*W’*s ≥ 0.94, *p’*s ≥ 0.30). A one-way repeated-measures analysis of variance (ANOVA) revealed a significant main effect of test expression, *F*(2,26) = 9.27, *p* = 0.001, $\eta_{p}^{2}$ = 0.42. *Post hoc* tests with Bonferroni correction showed higher recognition performance for neutral expression relative to happy expression and angry expression, *p’*s = 0.049 and 0.001, respectively. More interestingly, however, the performance for the happy expression condition was higher than the angry expression condition, *p* = 0.047.

We also calculated criterion (*c*), where *c* = -[z(H) + z(FA)]/2 (Stanislaw and Todorov, 1999). A negative value of *c* signifies a liberal, whereas a positive value a conservative bias. Results of *c* are shown in **Figure 1B**. The data were again normally distributed (*W’*s ≥ 0.89, *p’*s ≥ 0.08). ANOVA showed a significant main effect of test expression, *F*(2,26) = 31.96, *p* < 0.001, $\eta_{p}^{2}$ = 0.71. *Post hoc* tests showed that the response criterion was most liberal for neutral face (*p*’s < 0.001), most conservative for happy face (*p* < 0.001, *p* = 0.015), while the response criterion for angry face was in between (*p* = 0.015, *p* < 0.001).

**FIGURE 2:**
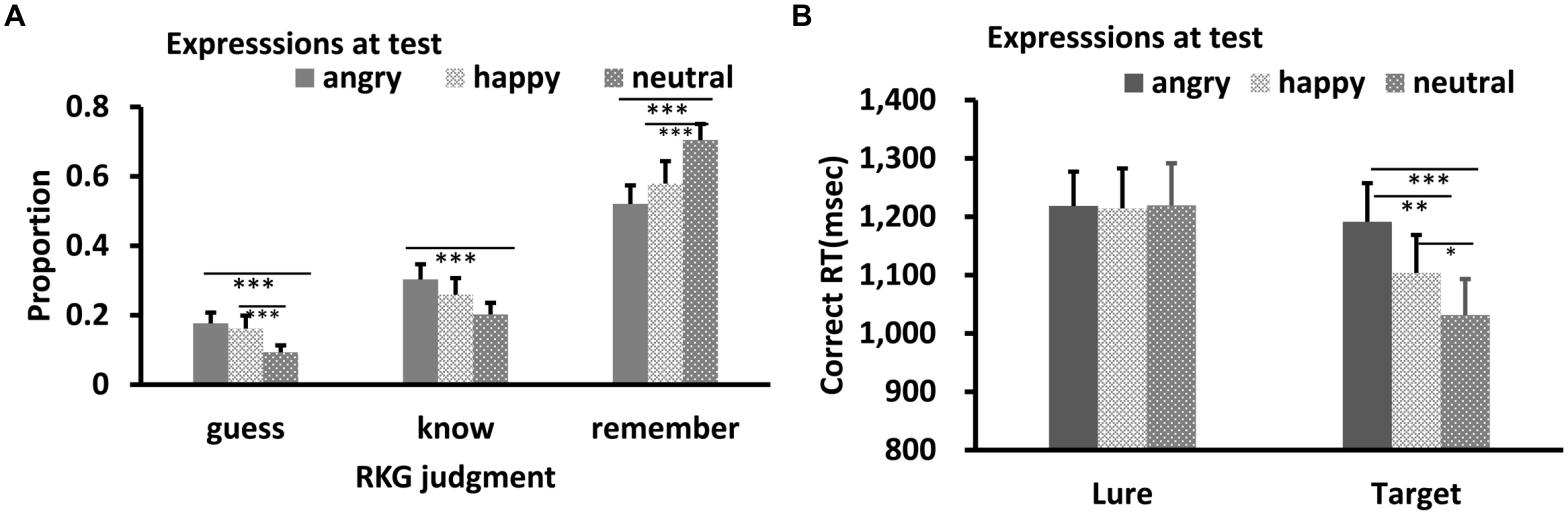
**RKG judgment (A) and Correct RT (B) as a function of test expression (Experiment 1)**. Error bars represent 1 SEM. ^∗^*p* < 0.05; ^∗∗^*p* < 0.01; ^∗∗∗^*p* < 0.001.

Results of RKG judgment of old faces are shown in **Figure 2A**. Shapiro–Wilk tests showed that the data in all conditions were normally distributed (*W’*s ≥ 0.90, *p’*s ≥ 0.11). Separate ANOVA was conducted for each of the three types of RKG judgments. All showed a significant main effect of test expression: Remember, *F*(2,26) = 20.81, *p* < 0.001, $\eta_{p}^{2}$ = 0.62; Know, *F*(2,26) = 5.66, *p* = 0.009, $\eta_{p}^{2}$ = 0.30; Guess, *F*(2,26) = 7.87, *p* = 0.002, $\eta_{p}^{2}$ = 0.38. *Post hoc* tests showed that the “remember” rate for neutral face was higher than for happy or angry faces, *p’*s < 0.001, and the “remember” rate for happy face was marginally higher than for angry face, *p* = 0.069. The guess rate for neutral face was lowest, *p* = 0.001, 0.007, while “guess” rates for happy and angry faces were comparable, *p* = 0.58. The “Know” rate for angry faces was higher than for neutral faces, *p* = 0.006. Results of other pair-wise comparisons were not significant.

Results of reaction times for correct responses are shown in **Figure 2B**. The data were normally distributed (*W’*s ≥ 0.92, *p’*s ≥ 0.29). ANOVA showed significant main effects of target-lure, *F*(1,13) = 7.34, *p* = 0.019, η_p_^2^ = 0.38 and test expression, *F*(2,26) = 8.97, *p* = 0.001, $\eta_{p}^{2}$ = 0.43. The interaction between these was also significant, *F*(2,26) = 6.73, *p* = 0.005, η_p_^2^ = 0.36. *Post hoc* tests showed no significant RT difference among the three test expression for lures, *p*’s = 0.87, 0.89, and 0.97, respectively. However, RT was slowest for angry targets relative to happy and neutral targets, *p*’s = 0.009 and <0.001, respectively. Response for the happy targets were slower than for neutral targets, *p* = 0.026.

Because image similarity between the learning-test pairs was measured by SSIM, it was possible to control for the influence of this had on memory performance by including it as a covariate in an item-based analyses of covariance (ANCOVA). One of the assumptions of ANCOVA is that the covariate must be linearly related to the dependent variable. Hence we first tested whether this assumption was met. We only found the image similarity scores correlated significantly with hit rates, remember rates and hit RTs, *r* = 0.49, 0.51, -0.49, respectively, all *p’*s < 0.001. ANCOVA for hit rates and hit RTs showed a non-significant effect of image similarity, *F’*s(1,104) = 0.26, 1.41, *p’*s ≥ 0.61, 0.23, $\eta_{p}^{2}$ = 0.002, 0.013, and the effect of test expression remained significant, *F*(2,104) = 4.34, 7.09, *p’*s ≥ 0.015, 0.001, $\eta_{p}^{2}$ = 0.077, 0.12, respectively. ANCOVA for remember rates showed a significant effect of the similarity, *F*(1,104) = 4.30, *p* = 0.04, $\eta_{p}^{2}$ = 0.04, and the effect of test expression remained significant, *F*(2,104) = 8.22, *p* < 0.001, η_p_^2^ = 0.14.

To recap, the key finding in this experiment was that the performance for the angry expression condition was poorer than for the happy expression condition (lower sensitivity and remember rate, slower RT). Further analysis based on the similarity measure ruled out the alternative explanation that this finding was due to a physical image similarity between the training and test images.

## Experiment 2

Unlike Experiment 1, where neutral faces were used as learning stimuli, prior studies often chose emotional faces as learning stimuli. However, because the test stimuli in these studies were often identical to the learning stimuli, it is not possible to judge whether the reported effect of facial expression depended on an association between the emotional content of the face images at the stages of encoding and retrieval. To investigate this issue, we ran Experiment 2 using angry or happy faces as learning stimuli. We assessed the effect of expression as retrieval cues after learning faces with a happy (Experiment 2a) or angry (Experiment 2b) expression. Our key interest was to find out whether after studying a face with a happy expression, the identity of the face could be recognized more effectively when the test faces display a neutral or an angry expression (Experiment 2a), or whether after studying a face with an angry expression, recognition of the face could be more successful when the test faces display a neutral or happy expression (Experiment 2b). In both cases, the facial expressions displayed at learning (encoding) and test (retrieval) formed no obvious emotional association. This allowed us to further assess whether any effect of expression on identity recognition could be independent of emotional association between learning and test stimuli.

### Method

#### Participants

Sixteen undergraduate students from Beijing Forestry University (mean age 22.6 years, SD = 1.9, 8 females) participated in Experiment 2a. Fourteen undergraduate students from Chinese Agricultural University (mean age 23.4 years, SD = 3.3, 9 females) participated in Experiment 2b. All participated for a small payment, and all had normal or correct-to-normal vision. Informed consent was obtained from each participant prior to the experiment.

#### Materials

The face stimuli were identical to Experiment 1.

#### Design and Procedure

These were identical to Experiment 1, except that learning stimuli were happy faces in Experiment 2a and angry faces in Experiment 2b.

### Results and Discussion

**FIGURE 3:**
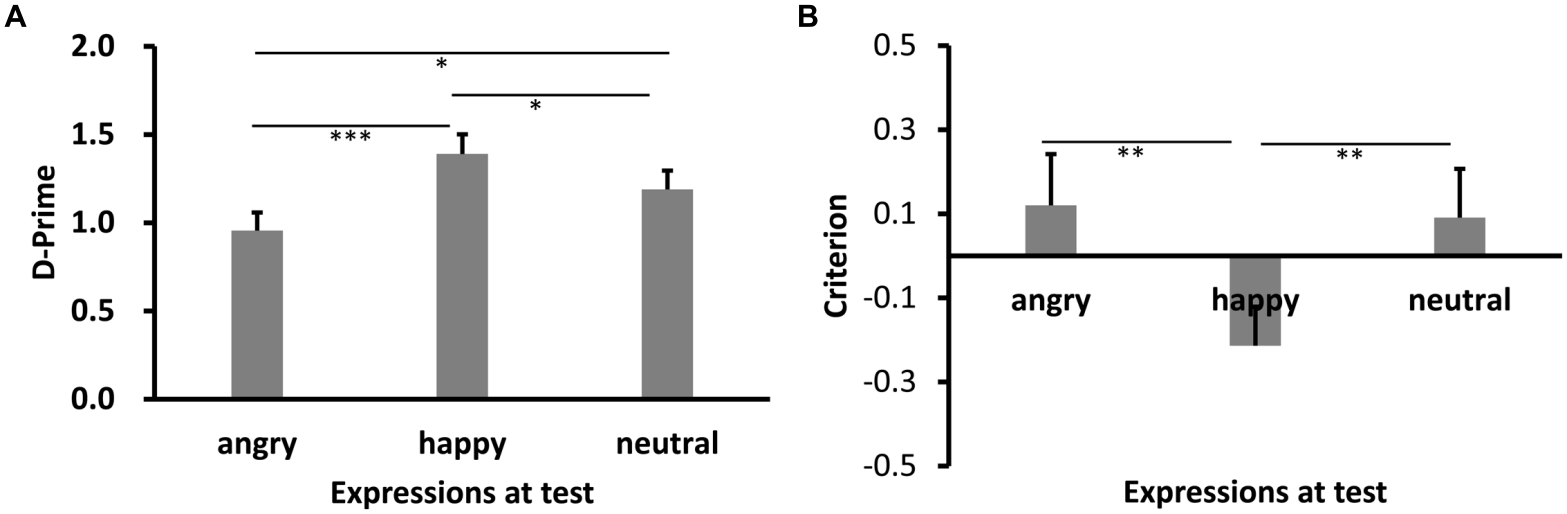
**Sensitivity *d*′ (A) and Criterion c (B) results as a function of test expression (Experiment 2a)**. Error bars represent 1 SEM. ^∗^*p* < 0.05; ^∗∗^*p* < 0.01; ^∗∗∗^*p* < 0.001.

**FIGURE 4:**
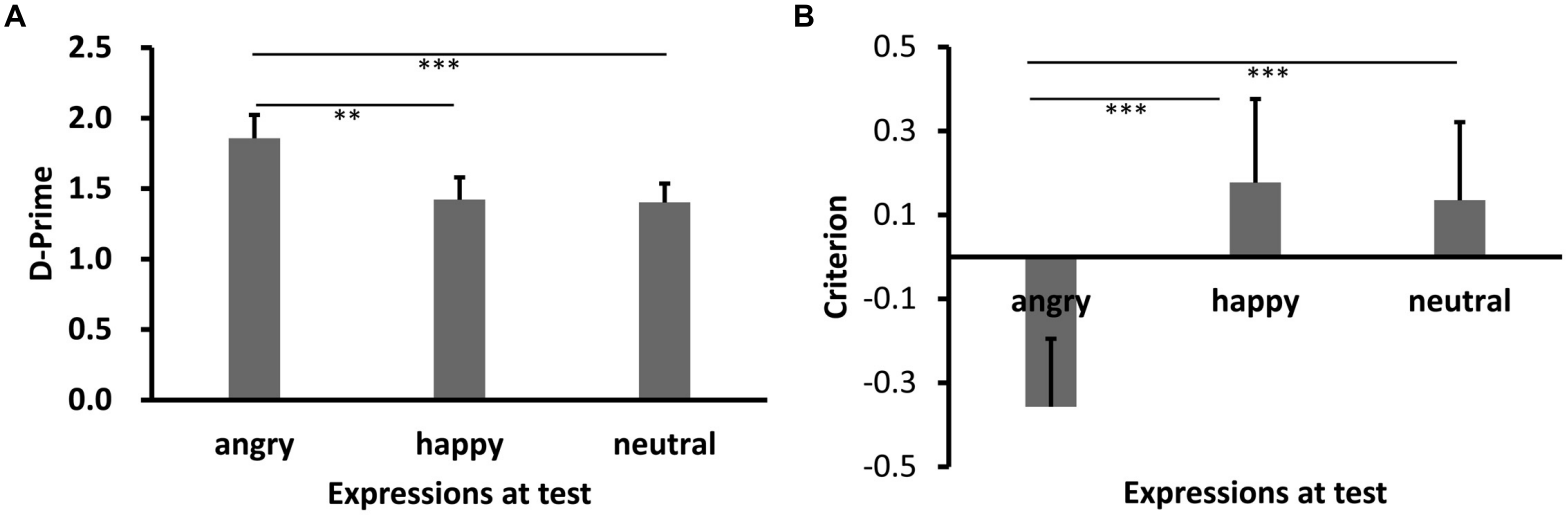
**Sensitivity *d*′ (A) and Criterion c (B) results as a function of test expression (Experiment 2b)**. Error bars represent 1 SEM. ^∗∗^*p* < 0.01; ^∗∗∗^*p* < 0.001.

Results of *d′* are shown in **Figures 3A and 4A** (Experiments 2a,b). Shapiro–Wilk tests showed that the data in all conditions were normally distributed (*W’*s ≥ 0.92, *p’*s ≥ 0.27). Both showed significant main effects of test expression, *F’*s = 11.93 (2a), 10.52 (2b), *p’*s < 0.001, $\eta_{p}^{2}$ = 0.45, 0.44, respectively. *Post hoc* tests showed higher recognition performance for same learning-test expression condition relative to different learn-test expression, *p’*s < 0.001 (2a: happy vs. angry), *p’*s = 0.045 (2a: happy vs. neutral), 0.003(2b: angry vs. happy), and 0.001 (2b: angry vs. neutral). Importantly, the performance for the angry expression condition was lower than the neutral expression condition in Experiment 2a, *p* = 0.017, while the performance for the happy expression condition was comparable to the neutral expression condition in Experiment 2b, *p* = 0.85.

Results of *c* are shown in **Figures 3B and 4B**. The data in all conditions were normally distributed (*W’*s ≥ 0.94, *p’*s ≥ 0.41). There was a significant main effect of test expression in Experiment 2a, *F*(2,30) = 11.59, *p* < 0.001, $\eta_{p}^{2}$ = 0.44. *Post hoc* tests showed that the response criterion was more liberal for same learning-test expression than different learn-test expressions [*p*’s = 0.002 (happy vs. angry), and 0.003 (happy vs. neutral), respectively], while the response criterion for the two different learn-test expression conditions were comparable (*p* = 0.55, angry vs. neutral). The main effect of test expression in Experiment 2b was also significant, *F*(2,26) = 30.02, *p* < 0.001, $\eta_{p}^{2}$ = 0.70. *Post hoc* tests showed that the response criterion was more liberal for same learning-test expression than different learning-test expression (*p*’s < 0.001, angry vs. happy, and angry vs. neutral), while the response criterion for the two different learning-test expression conditions were comparable (*p* = 0.53, happy vs. neutral).

**FIGURE 5:**
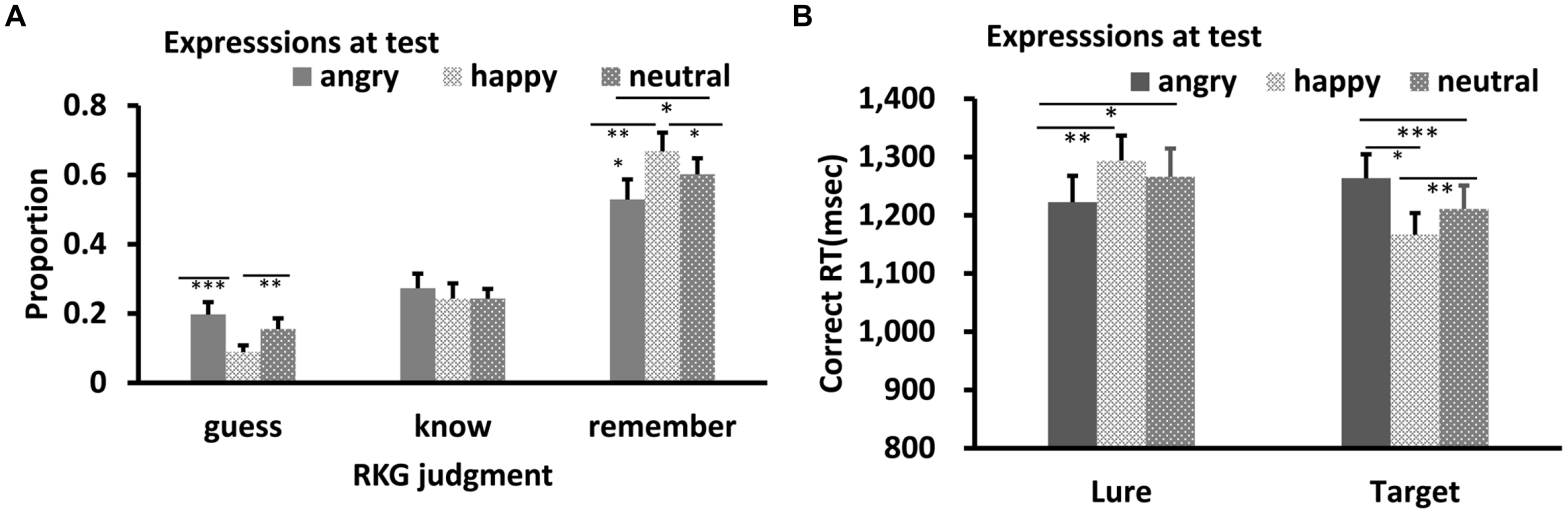
**RKG judgment (A) and Correct RT (B) as a function of test expression (Experiment 2a)**. Error bars represent 1 SEM.^∗^*p* < 0.05; ^∗∗^*p* < 0.01; ^∗∗∗^*p* < 0.001.

**FIGURE 6:**
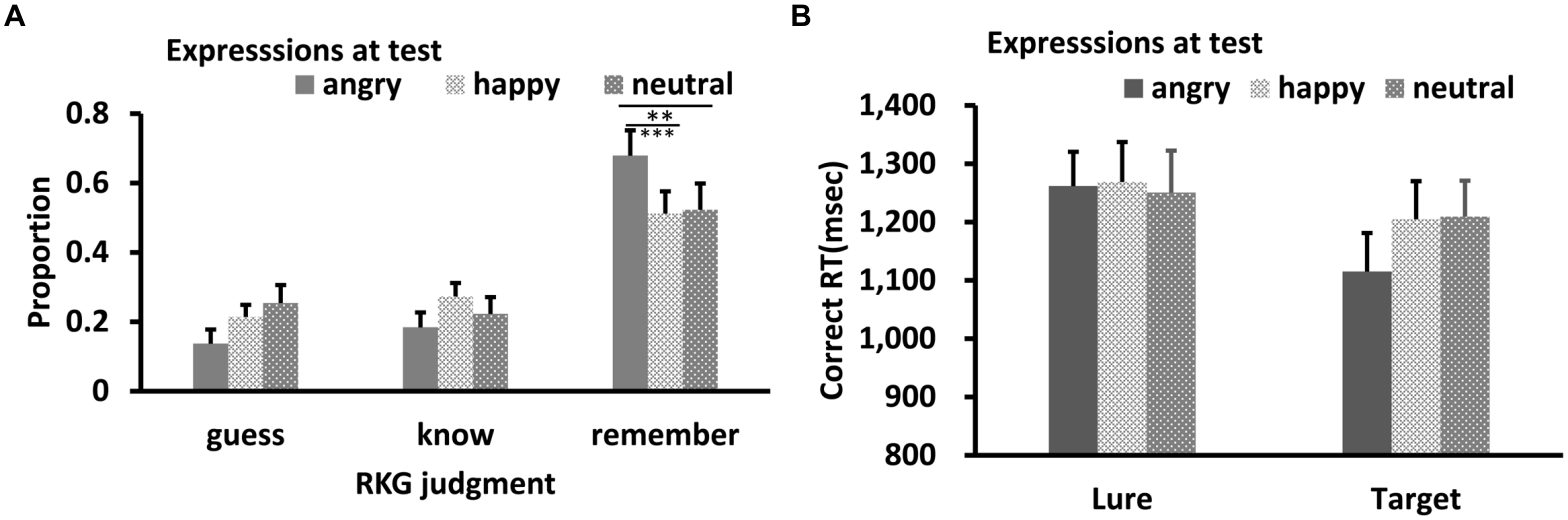
**RKG judgment (A) and Correct RT (B) as a function of test expression (Experiment 2b)**. Error bars represent 1 SEM. ^∗∗^*p* < 0.01; ^∗∗∗^*p* < 0.001.

Results of RKG judgment of “old” faces are shown in **Figures 5A and 6A**. The data in all conditions were normally distributed (*W’*s ≥ 0.89, *p’*s ≥ 0.05). ANOVA showed a significant main effect of test expression for “Remember” judgment in both Experiments 2a,b, *F’*s = 10.08, and 13.15, *p’*s < 0.001, $\eta_{p}^{2}$ = 0.40, and 0.50, respectively. In Experiment 2a, *post hoc* test showed a higher “remember” rate for happy test faces than for angry (*p* < 0.001) and neutral test faces (*p* = 0.05), a lower “remember” rate for angry test faces than for neutral test faces, *p* = 0.037, and a lower “guess” rate for happy test faces than for angry (*p* < 0.001) or neutral test faces (*p* = 0.007), while the “guess” rates of angry and neutral test faces were comparable, *p* = 0.10. In Experiment 2b, “remember” rate for angry test faces was higher than for happy (*p* < 0.001) and neutral test faces (*p* = 0.007), while “remember” rate of happy and neutral test faces were comparable, *p* = 0.51. No significant effect for “Know” judgment in Experiment 2a, *F* = 0.78, *p* = 0.47; no significant effect for “Know” and “Guess” judgment in Experiment 2b, *F’*s = 0.73, and 1.81, *p’*s = 0.49, and 0.18.

For Correct RT in Experiment 2a (**Figure 5B**), Shapiro–Wilk tests showed that the data were normally distributed (*W*’s > 0.91, *p*’s > 0.13) in all conditions, except for the happy lures and angry targets condition (*W*’s < 0.87, *p*’s < 0.03). ANOVA showed no significant main effect for target-lure, *F*(1,15) = 2.72, *p* = 0.12, η_p_^2^ = 0.15, or test expression, *F*(2,30) = 0.40, *p* = 0.67, η_p_^2^ = 0.03. However, this was qualified by a significant interaction, *F*(2,30) = 22.55, *p* < 0.001, η_p_^2^ = 0.60. *Post hoc* tests showed quicker RT for angry lure than for happy (*p* = 0.003) or neutral lure (*p* = 0.046). RT for happy and neutral lures were comparable, *p* = 0.097. However, response for angry targets was slower relative to both happy (*p* < 0.001) and neutral targets (*p* = 0.027), and response for neutral targets was slower than happy targets, *p* = 0.005.

For Correct RT in Experiment 2b (**Figure 6B**), Shapiro–Wilk tests showed that the data in all conditions were normally distributed (*W*’s > 0.91, *p*’s > 0.19). ANOVA showed no significant main effect for target-lure, *F*(1,13) = 0.007, *p* = 0.94, η_p_^2^ = 0.001, or test expression, *F*(2,26) = 2.39, *p* = 0.12, η_p_^2^ = 0.18. The interaction between these was also not significant, *F*(2,26) = 1.74, *p* = 0.20, η_p_^2^ = 0.14.

The key finding in this experiment was a detrimental effect of angry face cue on identity retrieval after the faces were learned with a happy expression. There was also a robust effect of happy face cue on identity retrieval after the faces were learned with an angry expression. Taken together, the results suggest that an angry test face impairs retrieval regardless of the facial expression displayed at the time of encoding.

## General Discussion

This study aimed to investigate the effect of emotional facial expression as a retrieval cue on long-term memory of a face identity. It also investigated whether facial expression affects identity recognition when an emotional association between learning and test stimuli is absent. Results in Experiment 1 showed a clear emotional cue effect, where a face identity was recognized more accurately when the test face showed a happy expression relative to an angry expression. Experiment 2 further showed that this emotional cue effect is robust across different encoding conditions where faces were learned with happy or angry expressions. Both experiments demonstrated that the effect of emotional expression on identity recognition does not rely on emotional association between learning and test faces.

### Influence of Emotional Cues on Face Recognition

Prior research suggested that emotion can consolidate and strengthen memory trace of learned faces, which explains more efficient memory retrieval (Mickley and Kensinger, 2008). Our data in Experiment 1 further showed that effect of facial expression can also be demonstrated at the stage of retrieval without relying on the influence of expression at the stages of encoding and consolidation. In Experiment 2, we found that the recognition performance of happy faces was lower when the retrieval cue was an angry face relative to a neutral face, whereas a happy face cue and a neutral face cue led to comparable recognition performance of angry faces. Hence the effect of expression on identity processing is not limited to the strengthened long-term memory trace.

What is the cause for this advantage of positive emotional cue or the disadvantage of negative emotional cue? Although explanations could be sought either from an appraisal of emotional valence or from physical features of the emotional faces, the method and analysis in this study rule out the physical account. If identity recognition performance could be predicted from image similarity between the learning and test stimuli, an angry face cue should have produced better performance in Experiment 1, because of its higher similarity with neutral faces presented at learning. The results, however, showed a happy face advantage instead. Our analysis of covariate provided further support that image similarity cannot account for the difference between effects of happy and angry face cues.

Thus the advantage of positive emotional cue is more likely to be mediated by an appraisal of emotional information. It is well known that positive emotions lead to greater holistic processing of faces, whereas negative emotions are linked with feature-based processing (Curby et al., 2012). Given that memory representation of face is holistic (Chan and Ryan, 2012; Heptonstall et al., 2013), a test face with a happy expression should facilitate, whereas a test face with an angry expression should impair the retrieval process in an identity recognition task. Our results were consistent with this interpretation. However, it should be noted that the evidence from the present study for this interpretation was found in the retrieval phase. This differred from previous studies that used identical emotional stimuli at learning and test. The facilitating effects in these studies may have resulted from encoding-retrieval emotional correspondence (Danker and Anderson, 2010) and/or a consolidation process (McGaugh, 2004; Schmidt and Saari, 2007; Righi et al., 2012). It is unlikely that the beneficial effect of happy faces at retrieval in the present study was due to memory consolidation, because our results show that the effect could occur without seeing a happy expression at the time of encoding.

It has been thought previously that positive emotions create increased feelings of familiarity, whereas negative ones lead to a more vivid recollection (Mickley and Kensinger, 2008). However, this is far from conclusive. There is limited evidence for the hypothesis from neural data, without measurable difference from behavioral data (Mickley and Kensinger, 2008). Our data also produced no support for the hypothesis, as we found no difference between feelings of familiarity for happy and angry faces. In fact, we found that happy faces were more vividly recollected than angry faces. A potential explanation could be related to the task characteristics: the learning task was intentional in this study, but incidental in Mickley and Kensinger (2008). D’Argembeau et al. (2003) suggest that intentional learning engages a higher degree of conscious elaboration at study than incidental learning and thereby enhances “Remember” but does not affect “Know” responses. Consistent with this hypothesis, their participants showed a tendency to make more “Remember” responses for happy than for angry faces in the intentional, but not in the incidental, learning condition. Our data are consistent with their finding, and further suggest that a positive expression can facilitate recollection without an emotional association between stimuli used at learning and test. This is different from the effect of emotional stimuli on encoding and consolidation, which may be a result of an emotional association between the learning and test conditions.

### Influence of Emotional Cues on Recognition Bias

In addition to the modulation of emotion on memory matching accuracy, face memory is also modulated by the facial expression via response bias. Sergerie et al. (2007) used an incidental memory task with emotional faces. They found a liberal bias (tendency to say “old”) for negative faces and conservative bias (tendency to say “new”) for positive faces. Our results were consistent with the common findings of liberal bias for negative stimuli, but inconsistent with the results in Phaf and Rotteveel (2005), who used emotional information as stimulus context. However, it is unclear whether this bias is independent of the valence of the face stimuli at the time of encoding, because prior research employed identical face images in the learning and test sessions. The present study was able to show that the criterion shift can be independent of the effect of encoding and consolidation, because Experiment 1 showed a clear conservative criterion for happy faces than for angry faces when faces were encoded with a neutral expression. Similar to Sergerie et al. (2007) our analysis of the relationship between image similarity and response bias suggest that this emotion-dependent response bias is unlikely due to perception of simple featural similarities (e.g., angry faces being more similar to neutral faces and thus resulting in a higher confusion between the old and new faces).

### The Role of Image Similarity in Face Memory

Although there was a clear emotional cue effect when expression changed between learning and test, all the three experiments showed a cost of this image change compared to baseline condition with identical images. This is a common observation in the literature, and it follows the idea that the accuracy of recognition memory is a function of encoding-retrieval match (Tulving and Thomson, 1973; Ritchey et al., 2013). Similar cost of expression transfer has been reported in tasks involving either long-term memory (e.g., D’Argembeau and Van der Linden, 2007; Righi et al., 2012) or short-term memory (e.g., Chen and Liu, 2009; Chen et al., 2011). The present study shows again that similarity between learned and tested images contributes to recognition performance. However, the main point of this study is that when facial expression is changed between learning and test, image similarity alone cannot explain identity recognition.

In summary, this study demonstrated a clear influence of emotional expression on identity recognition at the time of memory retrieval. Our method allowed us to separate the effect of an emotional expression on retrieval from the effect of the emotional expression on encoding and consolidation. We found that the retrieval cue effect on identity recognition is not only subject to image similarity between learned and tested faces, but also modulated by the emotional expression of the test face.

## Conflict of Interest Statement

The authors declare that the research was conducted in the absence of any commercial or financial relationships that could be construed as a potential conflict of interest.
